# Chronic hepatitis C virus infection irreversibly impacts human natural killer cell repertoire diversity

**DOI:** 10.1038/s41467-018-04685-9

**Published:** 2018-06-11

**Authors:** Benedikt Strunz, Julia Hengst, Katja Deterding, Michael P. Manns, Markus Cornberg, Hans-Gustaf Ljunggren, Heiner Wedemeyer, Niklas K. Björkström

**Affiliations:** 1 Department of Medicine Huddinge, Center for Infectious Medicine, Karolinska Institutet, Karolinska University Hospital Huddinge, 14186 Stockholm, Sweden; 2 Department of Gastroenterology, Hepatology and Endocrinology, Hannover Medical School, 30625 Hannover, Germany; 3 German Center for Infection Research (DZIF), Partner Site Hannover-Braunschweig, Hannover Medical School, 30625 Hannover, Germany; 4 Helmholtz Center for Infection Research, 38124 Braunschweig, Germany; 5 Department of Gastroenterology and Hepatology, Essen University Hospital, 45147 Essen, Germany

## Abstract

Diversity is a central requirement for the immune system’s capacity to adequately clear a variety of different infections. As such, natural killer (NK) cells represent a highly diverse population of innate lymphocytes important in the early response against viruses. Yet, the extent to which a chronic pathogen affects NK cell diversity is largely unknown. Here we study NK cell functional diversification in chronic hepatitis C virus (HCV) infection. High-dimensional flow cytometer assays combined with stochastic neighbor embedding analysis reveal that chronic HCV infection induces functional imprinting on human NK cells that is largely irreversible and persists long after successful interventional clearance of the virus. Furthermore, HCV infection increases inter-individual, but decreases intra-individual, NK cell diversity. Taken together, our results provide insights into how the history of infections affects human NK cell diversity.

## Abstract (web summary)

Natural killer (NK) cells are important immune cells for mediating antiviral immunity. Here the authors show that chronic hepatitis C virus infection in human can imprint lasting functional phenotypes in NK cells to increase their inter-individual but decrease intra-individual diversity.

## Introduction

Natural killer (NK) cells are abundant innate lymphocytes that contribute to antiviral immune responses^1^. Unlike T and B cells that utilize somatically recombined antigen specific receptors, NK cells employ an array of germline-encoded activating and inhibitory receptors expressed on their surfaces when interacting with infected target cells^2^. The balance between signaling through these receptors determines the outcome of NK cell activation with ensuing effector responses^2^. NK cells were long thought to be a rather homogeneous population of cells with limited diversity and fixed functional, as well as phenotypic properties. However, a plethora of results from both mouse and human studies has revealed NK cells to be much more diverse than previously appreciated^3–6^.

Both genetic and environmental factors cooperate in generating large numbers of NK cell subpopulations with distinct characteristics^3,7^. Examples of factors contributing to high NK cell diversity include the NK cell differentiation process^8,9^, variegated and stochastic expression of killer cell immunoglobulin-like receptors (KIR)^10^, NK cell education^11,12^, and the influence of tissue microenvironments on the NK cell compartment^3^. Furthermore, the composition of NK cells can dynamically adapt over an individual’s lifetime primarily in response to encountered infections^3,13^. This is underscored by the appearance of adaptive-like NK cell expansions in individuals latently infected with cytomegalovirus (CMV)^14–16^. Additionally, chronic infections by viruses such as human immunodeficiency virus (HIV)-1 and hepatitis C virus (HCV) can promote the appearance of phenotypically and functionally abnormal CD56^neg^ NK cells^17,18^. Although previous work has characterized human NK cells in chronic viral infections to some extent, few studies have examined in depth the affection of the full spectrum of NK cell subpopulations upon an infection. In particular, it is still largely unknown whether changes inflicted by a chronic infection on the NK cell compartment are reversible upon resolution of the infection.

To address these questions, we set out to study human HCV infection. HCV is an extremely successful pathogen in terms of the ability to establish a chronic infection^19^. Moreover, genetic data and cellular studies indicate that NK cells have an important function in the defense against HCV^20–23^. Using high-dimensional flow cytometry coupled to an unsupervised analysis approach, as well as implementing novel metrics of immune system composition^24,25^, we show that chronic HCV infection has a significant effect on diversity of the human NK cell repertoire. The advent of highly effective direct-acting antivirals (DAA) has revolutionized HCV treatment in the last few years with most patients now clearing the infection within weeks after treatment^26^. By using this outcome as a model for rapid pathogen removal, we further examined the durability of the imprint inflicted by chronic HCV on the NK cell compartment. Our results provide a global and comprehensive view of how a chronic viral infection affects diversity of the human NK cell repertoire.

## Results

### Imprint by chronic HCV infection on the NK cell repertoire

**Fig. 1:**
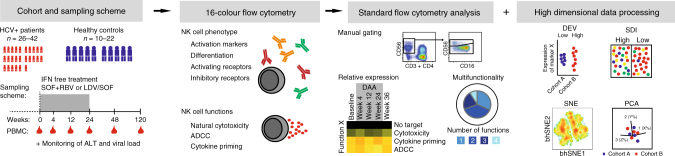
Study outline. In total, blood samples from 29 patients with chronic HCV were collected during treatment with direct-acting antivirals (before sofosbuvir + ribavirin (SOF + RBV) therapy (*n* = 26), at week 4 (*n* = 25), 12 (*n* = 24), 24 (*n* = 24), 36 (*n* = 21), and at long-term follow-up week 120 (*n* = 9), as well as from 22 matched healthy controls. Chronic HCV patients receiving ledipasvir/sofosbuvir (LDV/SOF) were sampled at baseline (*n* = 10), week 2 (*n* = 1), 8 (*n* = 2), 12 (*n* = 7), and follow-up weeks 36 (*n* = 2), 60 (*n* = 8), and 108 (*n* = 2). The NK cell phenotype and function was assessed using multi-color flow cytometry. These data were analyzed by conventional gating, relative expression of markers and multifunction, as well as in dimensionality reductive manners with stochastic neighbor embedding (SNE), principal component analysis (PCA), donor-to-donor expression variation (DEV), and inverse Simpson diversity index (SDI) analysis

**Fig. 2:**
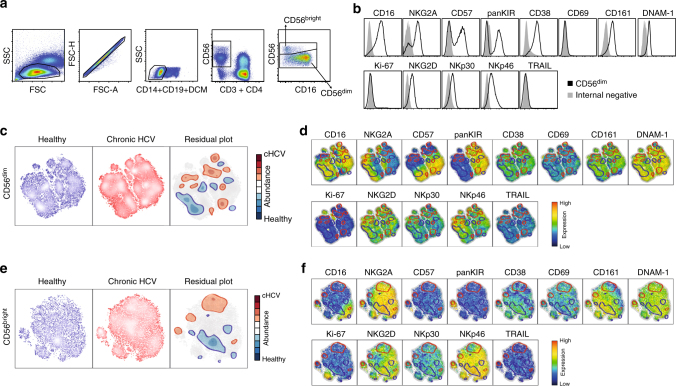
Imprint of chronic HCV on NK cells revealed by stochastic neighbor embedding analysis. **a**, **b** Representative staining for identification of CD56^bright^ and CD56^dim^ NK cells, as well as histograms for the NK cell phenotypic markers used in the SNE analysis. **c** SNE-plots of CD56^dim^ NK cells from healthy controls (*n* = 8) and patients with chronic HCV (*n* = 22), as well as a residual plot showing the difference between healthy subjects and those with chronic HCV. **d** SNE-plots showing expression intensities of the indicated markers for the 13 parameters of the SNE analysis in (**c**). Cellular events within blue circles are more abundant in healthy controls, whereas cellular events within red circles are enriched during chronic HCV infection. **e**, **f** Similar analysis as above but for CD56^bright^ NK cells (*n* = 20 for HCV patients and *n* = 8 for healthy controls)

To overcome the relative shortcoming of former studies of NK cells in the context of chronic viral infections typically probing simultaneously for only a limited number of phenotypic parameters, we here combined a high-dimensional flow cytometry analysis with stochastic neighbor embedding (SNE) analysis to determine the overall impact of chronic HCV on NK cells (Fig. 1). Thirteen inhibitory and activating receptors, as well as differentiation and activation markers, were simultaneously assessed on CD56^bright^ and CD56^dim^ NK cells at the single-cell level from ten healthy controls and 26 patients with chronic HCV infection (Fig. 2a, b, Supplementary Fig. 1, Supplementary Table 1). The data were electronically barcoded, merged, analyzed using Barnes-Hut SNE, and deconvoluted into SNE maps for patients and controls (Fig. 2c–f). Next, we subtracted individual population intensities of the control SNE-map from the patient SNE-map to visualize the specific NK cell traits of the HCV patients (Fig. 2c, e, Residual plot). To interpret these differences, results from the residual plots were projected onto additional SNE maps showing expression of the 13 individual parameters that were part of the analysis (Fig. 2d–f). This strategy revealed HCV-specific clusters expressing low levels of NKG2A, CD16, NKp46, NKG2D, and DNAM-1. Importantly, some of these differences could be recapitulated by using conventional single-parameter flow cytometry analysis (Supplementary Fig. 1). However, other differences were present only in multi-dimensional space, as revealed by the SNE analysis. Together, these results provide a comprehensive high-dimensional map of the circulating human NK cell compartment in healthy individuals and in patients with chronic HCV infection. The results also reveal considerable differences amongst the two groups.

### Validation of metrics gauging NK cell repertoire diversity

Since individual NK cell receptors are expressed in a variegated fashion on single cells, the human NK cell repertoire is highly diverse both within and between individuals^6,25^. Although our SNE analysis of HCV patients substantiated previously described phenotypic alterations^27,28^, as well as identified novel differences, it did not provide a global estimate of how a chronic infection, such as HCV, perturbed the human NK cell repertoire. To address this issue, we employed recently described metrics of immune history and function, the inverse Simpson diversity index (SDI)^6,25^, as well as an algorithm to determine protein-expression variation (donor-to-donor expression variation (DEV)), which we adjusted from a recent report^24^.

**Fig. 3:**
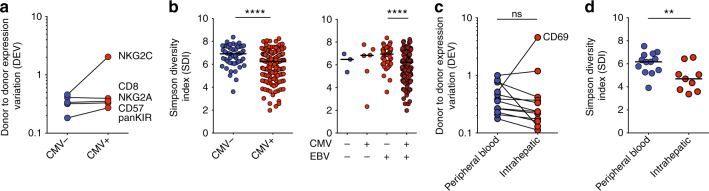
Validation of metrics assessing NK cell inter- and intra-individual diversity. **a**, **c** Donor-to-donor expression variation (DEV) and **b**, **d** inverse Simpson diversity index (SDI) were calculated as described for a cohort of healthy individuals (*n* = 202) grouped according to (**a**, **b**) CMV and **b** EBV serostatus, as well as (**c**, **d**) in intrahepatic NK cells (*n* = 9) compared with peripheral blood NK cells from healthy controls (*n* = 13). Mann–Whitney (SDI) and Wilcoxon test (DEV) were used for statistical analysis. Bars indicate median values, *p < 0.05 and **p < 0.01, ****p < 0.0001

We validated these measurements in two ways. First, by examining a large cohort of 202 healthy blood donors stratifying for CMV serostatus, which is known to affect the NK cell repertoire; and secondly by comparing the NK cell subset repertoire composition in peripheral blood with that of the liver. When assessing DEV in 202 healthy individuals and grouping for CMV serostatus, CMV seropositivity yielded higher DEV values for NKG2C and panKIR (Fig. 3a). This is in line with CMV promoting adaptive-like expansions that often express NKG2C and KIRs, and thus causing the observed increased inter-individual diversity^16^. When measuring SDI, we focused on NK cell differentiation. This process can be mapped using well-described phenotypic characteristics including the transition from CD56^bright^ to CD56^dim^ NK cells, as well as increased expression of CD57 and KIRs and loss of NKG2A within the CD56^dim^ compartment^3,8^. Indeed, and also as a sign of adaptive-like expansions, CMV, but not EBV, infection was able to cause a significant reduction in SDI (Fig. 3b, *p* < 0.0001 by Mann–Whitney test). As second validation, peripheral and intrahepatic NK cells were compared, as it is known that the latter have a distinct phenotype with for instance the presence of a sizeable fraction of CD56^bright^CD16^−^CD69^+^ tissue-resident NK cells exhibiting limited phenotypic diversity^29^. DEV was unaffected in the liver, except for CD69, which was highly increased (Fig. 3c). Instead, and as expected in line with exhibiting a more limited phenotypic diversity, intrahepatic NK cells had a considerably lower SDI as compared to peripheral blood NK cells (Fig. 3d).

Taken together, this shows that SDI and DEV are immune cell metrics examining inter- and intra-individual variation of the total NK cell compartment.

### Disturbed NK cell repertoire diversity in HCV infection

**Fig. 4:**
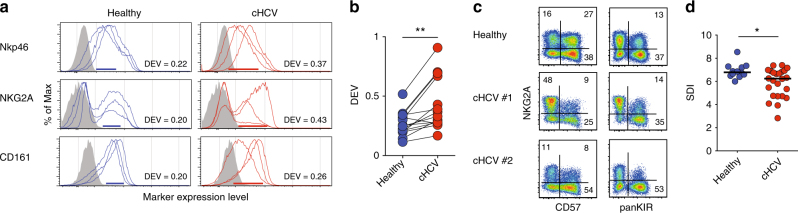
Disturbance of NK cell repertoire diversity in chronic HCV infection. **a** Histograms for NKp46, NKG2A, and CD161 expression for healthy controls and patients with chronic HCV. Histograms shown represent individuals in the 10^th^, median, and 90^th^ percentile from the respective cohorts. The donor-to-donor expression variation (DEV) for each of these markers is illustrated with blue and red bars in the histograms, as well as with the figures given. **b** Summary of data for DEV analyzed for 14 surface receptors in healthy controls (*n* = 10) and patients with chronic HCV (*n* = 24) . **c** Representative staining for NKG2A, CD57, and panKIR from one healthy control and two patients with chronic HCV. Figures within plots show frequency of positive cells within the respective quadrants. **d** Inverse Simpson diversity index (SDI) analysis of the Boolean-gates of CD56^bright^ NK cells as well as NKG2A, CD57, and panKIR expression within CD56^dim^ NK cells for healthy controls (*n* = 12) and patients with chronic HCV (*n* = 24). Mann–Whitney (SDI) and paired *t*-test (DEV) were used for statistical analysis. Bars represent median values, *p < 0.05, **p < 0.01

Having validated DEV and SDI as relevant metrics of variation within the NK cell compartment, we next examined patients with chronic HCV. Intriguingly, these patients had a significantly higher DEV as compared to healthy controls (Fig. 4a, b, *p* < 0.01 by paired *t*-test). Patients with chronic HCV infection also presented with significantly lower SDI as compared to the healthy controls (Fig. 4c, d, *p* < 0.05 by Mann–Whitney test). In contrast to chronic HCV infection, no such differences in DEV and SDI were observed when studying a cohort of patients with acute symptomatic HCV infection (Supplementary Fig. 2a, b). To connect NK cell repertoire diversity with the phenotypic differences observed in the SNE analysis, chronic HCV patients with an SDI more similar to healthy controls presented with fewer phenotypic alterations in the SNE analysis as compared to patients with low SDI-values (Supplementary Fig. 2c, d). Thus, applying two novel global estimates of immunological health to the human NK cell compartment yielded results showing that chronic HCV infection leads to increased DEV, as well as to decreased population diversity as measured by the SDI within an individual.

### Altered NK cell DEV associates with extent of liver disease

**Fig. 5:**
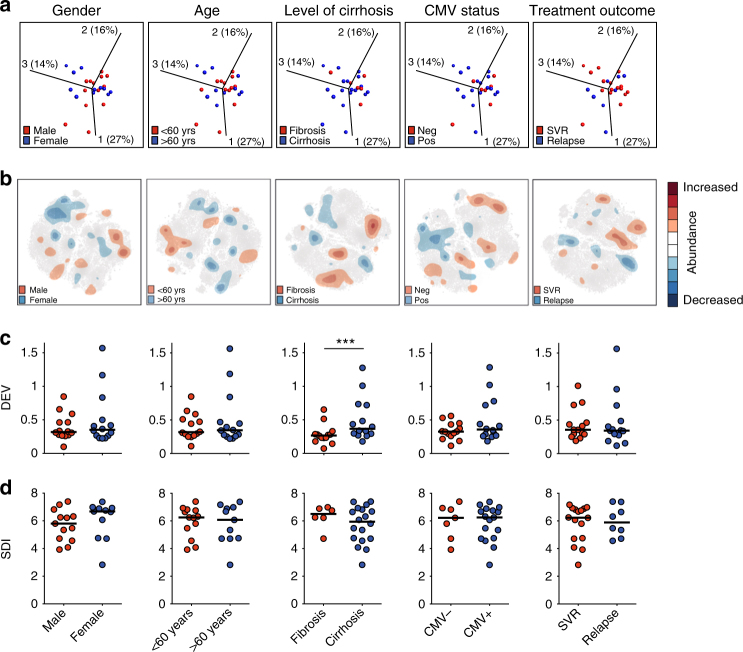
Influence of clinical parameters on NK cell repertoire diversity. **a** Principal component analysis (PCA), **b** stochastic neighbor embedding (SNE), **c** donor-to-donor expression variation (DEV), and **d** inverse Simpson diversity index (SDI) analyses of NK cells from patients with chronic HCV relative to clinical parameters. PCA and SNE analyses were performed on CD56^dim^ NK cells, DEV, and SDI on total NK cells. For gender, male *n* = 13, female *n* = 11; for age, <60 years *n* = 13, >60 years *n* = 11; for cirrhosis-status (cirrhosis defined as Fibroscan value > 14.5), fibrosis *n* = 6, cirrhosis *n* = 18; for CMV status, seronegative *n* = 7, seropositive *n* = 17; for treatment outcome, sustained virological response (SVR) *n* = 16, relapse *n* = 8. For SNE analysis, two samples were excluded due to different staining panels and one sample since it represented a significant outlier. Unpaired *t*-test, Mann–Whitney test (SDI), and Wilcoxon test (DEV) were used for statistical analysis. In (**c**) and (**d**), bars represent median values, ***p < 0.001

Having concluded that chronic HCV infection affects the human NK cell compartment in multiple yet distinct ways, we next analyzed whether these alterations covariate with either gender, age, extent of liver disease, antiviral treatment response, CMV serostatus, HCV genotype, or *KIR-HLA* genotypes. To that end, we performed SNE, DEV, and SDI analysis, as well as a principal component analysis (PCA) on NK cells from the 26 HCV patients after stratifying for the clinical, virological, and genetic parameters specified. Results from the PCA analysis showed no relevant clustering of distinct phenotypes when stratifying for age, gender, extent of liver disease, CMV seropositivity, or subsequent antiviral treatment outcome after treatment with sofosbuvir and ribavirin (SOF + RBV) (Fig. 5a, Supplementary Fig. 3a). To generate the PCA plots, *p*-values were set to 1 and *q*-values to 0 since the individual patients were not otherwise amenable to depiction. Likewise, although SNE analysis showed minor differences in the residual plot analysis, these were not linked to distinct phenotypes (Fig. 5b, Supplementary Fig. 3a). The absence of significant differences could also be confirmed by conventional flow cytometry analysis (Supplementary Fig. 4). This property is in contrast to the distinct imprint of chronic HCV observed when compared with that from healthy controls (Fig. 2c–f, Supplementary Fig. 1). However, patients with liver cirrhosis (liver elastography determined by Fibroscan® > 14.5 kPa) had significantly higher DEVs than patients with milder liver disease (Fig. 5c, *p* < 0.001 by Wilcoxon test).

To further substantiate the association between degree of liver disease and NK cell diversity, we included two additional longitudinal patient cohorts, one with more severe liver disease (subsequently treated with SOF + RBV) and one with less severe liver disease (subsequently receiving antiviral treatment with ledipasvir and sofosbuvir (LDV/SOF)) (Supplementary Table 2). Also in these two cohorts, we could detect a link between severity of liver disease and DEV (Supplementary Fig. 3b). From the original analysis, the decreased NK cell SDI was not significantly different when comparing patients with and without cirrhosis (Fig. 5d). However, most of the SDI-low patients were in the cirrhosis group. Thus, we also explored this tentative association in the two additional cohorts and could indeed also associate SDI to the degree of liver disease (Supplementary Fig. 3c). When merging all the three cohorts and finally stratifying for CMV again, no difference in DEV or SDI was observed as a direct consequence of CMV seropositity (Supplementary Fig. 3d). This suggests that the alterations observed above were due to chronic HCV infection. Regarding HCV genotype, no clear differences in SDI could be observed when comparing patients infected with different genotypes. Finally, we investigated the influence of *KIR* and *HLA* genotypes on DEV and SDI (Supplementary Table 3). Except for individuals carrying *KIR2DL3* and *HLA-C1/C1* presenting with lower DEV as compared to others (Supplementary Fig. 3e, f), no additional associations between *KIR* and *HLA* genotypes and SDI or DEV could be detected.

Taken together, this data show that altered DEV, and to some extent SDI, within the NK cell compartment during chronic HCV correlate with the extent of liver disease.

### NK cell functional capacity in chronic HCV infection

**Fig. 6:**
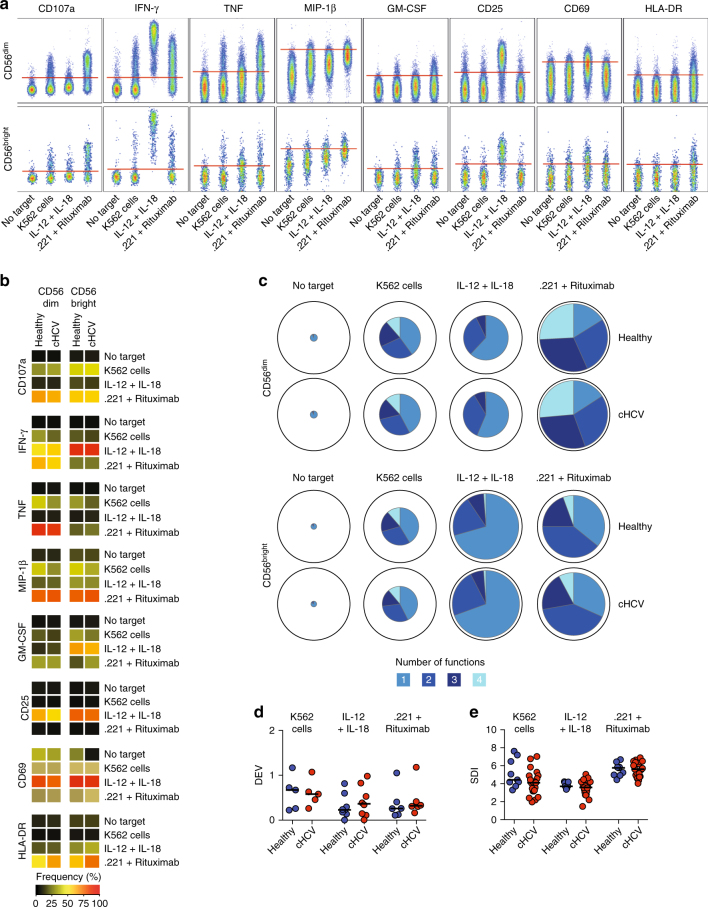
Functional capacity of NK cells in chronic HCV infection. **a** Representative staining for CD107a, IFN-γ, TNF, MIP-1β, GM-CSF, CD25, CD69, and HLA-DR within CD56^dim^ and CD56^bright^ NK cells after the indicated stimulations. No target represents the negative control. **b** Heatmap summarizing the median frequency of responding cells or median mean fluorescence intensity (MFI) (CD69 and MIP-1β) for the indicated functional readouts after stimulation for healthy controls (*n* = 9) and patients with chronic HCV (*n* = 21). **c** Multifunctional analysis of CD107a, IFN-γ, TNF, and MIP-1β responses for the indicated stimulation; color denotes the number of simultaneously exhibited functions. The median percentage of total responding cells for the functions is indicated by its size in the pie chart with total cells represented as the outer border. **d** Donor-to-donor expression variation (DEV) analysis of functional responses after the indicated stimulations. **e** Inverse Simpson Diversity Index (SDI) analysis based on CD107a, IFN-γ, TNF, and MIP-1β responses for the indicated stimulations. In (**d**) and (**e**), bars represent median values

Next, we assessed whether the phenotypic imprint observed would be recapitulated in the NK cells ability to perform natural cytotoxicity, respond to cytokines, or mediate ADCC. To examine these functions, we stimulated PBMC with either K562 cells, IL-12 + IL-18, or 721.221 cells coated with Rituximab. Eight functional measures (CD107a, IFN-γ, TNF, MIP-1β, GM-CSF, CD25, CD69, and HLA-DR) were then simultaneously evaluated on CD56^dim^ and CD56^bright^ NK cells, respectively (Fig. 6a). Unexpectedly, NK cells from patients with chronic HCV maintained their functional capacity; no major differences could be observed when these patients were compared with healthy controls (Fig. 6b, c). This was the case both when assessing single functional responses (Fig. 6b), as well as multifunctionality based on MIP-1β, CD107a, IFN-γ, and TNF responses (Fig. 6c). Similar results were obtained when we repeated the functional experiments in an additional cohort of patients with more severe liver disease (SOF + RBV, Supplementary Fig. 5a), whereas slightly increased functional responses were observed from a cohort with less severe liver disease (LDV/SOF, Supplementary Fig. 5a). Finally, when stratifying the original chronic HCV cohort into patients with low and normal phenotypic SDI (Supplementary Fig. 2c), low SDI was associated with reduced functional responses upon K562 stimulation within CD56^dim^ NK cells (Supplementary Fig. 5b). We also determined DEV and SDI of the actual functional responses (Fig. 6d, e). In contrast to the findings concerning NK cell phenotype, no significant alterations in NK cell functional DEV or SDI were observed in patients with chronic HCV as compared to the healthy controls.

### Persistent imprint on NK cell diversity upon HCV clearance

**Fig. 7:**
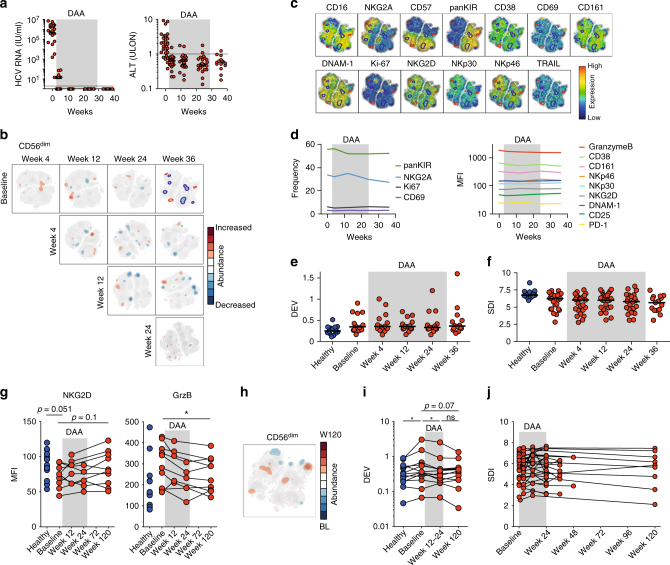
Long-lasting impact of chronic HCV infection on NK cell repertoire diversity after clearance of the virus. **a** HCV RNA and alanine transferase (ALT) levels in HCV patients before, during, and after treatment with direct-acting antivirals (DAA); dashed line indicates reference value. **b** Pairwise residual plots from stochastic neighbor embedding (SNE) analysis comparing the indicated time points for CD56^dim^ NK cells from patients with chronic HCV (*n* = 21-21-18-12 for the comparison baseline to week 4-12-24-36, *n* = 21-18-12 for week 4 to week 12-24-36, *n* = 17–12 for week 12 to week 24–35 and *n* = 11 for week 24–36). **c** The residual plot comparing baseline with week 36, which is shown in (**b**), projected onto individual density plots for the 13 markers included in the SNE analysis. **d** Summary of frequency of expression (left) and mean fluorescence intensity (MFI) (right) for the indicated receptors on NK cells from patients with chronic HCV shown at baseline and weeks 4, 12, 24, and 36 during and after treatment (*n* = 24, 24, 24, 21, and 15 for baseline, week 4, 12, 24, and 36). Median values are shown. **e**, **f** Summary of donor-to-donor expression variation (DEV) and inverse Simpson diversity index (SDI) analysis of NK cells from healthy controls (*n* = 10 for DEV and *n* = 12 for SDI) and patients with chronic HCV at baseline and weeks 4, 12, 24, and 36 during and after treatment (*n* = 24, 24, 24, 21, and 15 for these time points, respectively). **g** MFI of NKG2D (left panel) and Granzyme B (right panel) of CD56^dim^ NK cells from chronic HCV patients (*n* = 9) receiving sofosbuvir + ribavirin (SOF + RBV) assessed up to 120 weeks after start of treatment as compared to healthy controls (*n* = 13). **h** Residual plot from SNE analysis comparing baseline and 120 weeks after treatment. **i**, **j** DEV and SDI assessed up to 120 weeks after start of treatment (DEV, *n* = 9; SDI, *n* = 29). Unpaired or paired *t*-test as well as Wilcoxon test were used for statistical analyses. Bars represent median values, *p < 0.05

Treatment for HCV has undergone a revolution with the introduction of highly effective DAAs that rapidly eliminate the virus in almost all patients within weeks after treatment initiation^26^. We took advantage of this remarkable development and addressed whether the observed disturbances caused by HCV in the NK cell compartment were reversible upon rapid elimination of the virus. The cohort was sampled longitudinally during and directly after DAA treatment with SOF + RBV (Fig. 1). Treatment resulted in rapid decline of HCV RNA to undetectable levels as well as to normalization of ALT as a sign of reduced liver inflammation (Fig. 7a). For this cohort, SNE analysis was performed on NK cells at baseline and at weeks 4, 12, 24, and 36 after the start of DAA treatment. Then, pairwise comparisons of the SNE maps followed at the different time points (Fig. 7b, Supplementary Fig. 6a). In contrast to the clear imprint noted when comparing patients with chronic HCV to healthy controls (Fig. 2), only minor, if any, differences were observed over time in the patients (Fig. 7b, c, Supplementary Fig. 6a, b). This finding was confirmed by conventional single-parameter analysis (Fig. 7d). Of note, despite the high inter-individual variability among patients, the single marker expression was constant on an individual level (Supplementary Fig. 6c, d). As a consequence, the increased DEV and decreased SDI also remained stable throughout and directly after DAA-treatment (Fig. 7e, f).

A second cohort of patients on SOF + RBV treatment was sampled at very-long-term follow-up time points, up to 96 weeks after end of treatment (and thus 120 weeks after start of treatment). This gave us the opportunity to determine any long-lasting effects on NK cell repertoire diversity. Of the 15 markers assessed, we observed a normalization of Granzyme B expression at long-term follow-up and a trend towards restoration of NKG2D expression within CD56^dim^ NK cells (Fig. 7g), as well as a normalization of Granzyme B and NKp30 within CD56^bright^ NK cells. The observations were made after conventional flow cytometry gating and analysis. However, the pairwise SNE analysis between baseline and long-term follow-up, in line with the assessment directly after stop of treatment, presented with only minor phenotypic alterations (Fig. 7h). Of note, when assessing DEV, there was a trend towards normalization at more long-term follow-up time points (Fig. 7i). In contrast to this, SDI stayed suppressed up until two years after initial viral clearance (Fig. 7j).

SOF + RBV represents the first generation of DAAs from which a fraction of patients relapsed. Newer generations of DAAs are even more efficient with hardly any patients relapsing. To determine if NK cell repertoire diversity perhaps would become reinvigorated after a more efficient DAA regimen, we also assessed a cohort of patients receiving LDV/SOF over time. The 13-parameter SNE analysis revealed only minor differences comparing baseline with long-term follow-up time points (Supplementary Fig. 6e), and the same was evident after conventional flow cytometry analysis except for a slight reduction in CD38 within CD56^bright^ NK cells during and after DAA treatment. As mentioned above, the LDV/SOF cohort had less severe liver disease compared to the studied SOF + RBV groups (Supplementary Table 1 and 2). As such, no baseline difference was present in DEV as compared to healthy controls, and DEV also remained stable upon treatment and thereafter (Supplementary Fig. 6f). In contrast, SDI was low at baseline and remained suppressed up to 108 weeks after start of treatment (Supplementary Fig. 6g).

**Fig. 8:**
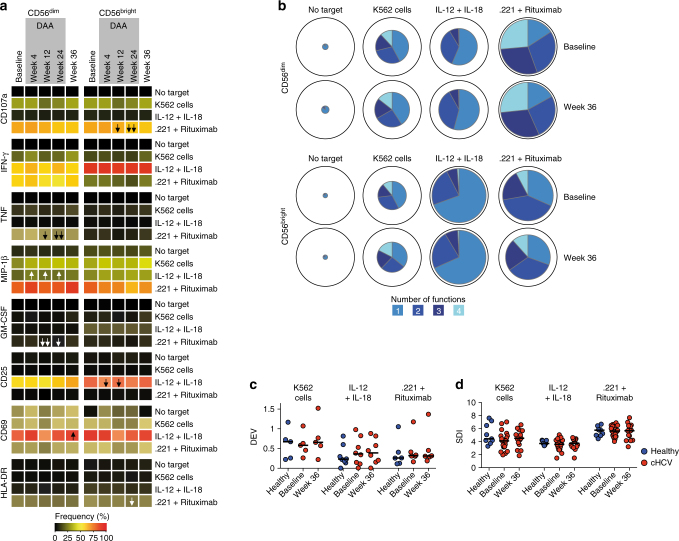
Minor modulations of NK cell function upon HCV clearance. **a** Heatmap summarizing the median frequency of responding cells or median mean fluorescence intensity (MFI) (CD69 and MIP-1β) for the indicated functional readouts after stimulation for chronic HCV patients (*n* = 21, 21, 20, 20, and 14 for baseline, week 4, 12, 24, and 36) at the indicated time points. One arrow (irrespective of color) in the heatmap represents *p* < 0.01, two arrows represent *p* < 0.001, the direction of arrows indicates an increased or decreased response as compared to baseline. **b** Multifunctional analysis of CD107a, IFN-γ, TNF, and MIP-1β responses of NK cells (*n* = 21 for baseline and *n* = 15 for week 36) for the indicated stimulations where the color denotes the number of simultaneously exhibited functions. The median percentage of total responding cells for each function is indicated by its size in the pie chart with total cells represented as the outer border. **c** Donor-to-donor expression variation (DEV) analysis of the function responses after the indicated stimulations. **d** Inverse Simpson Diversity Index (SDI) analysis based on CD107a, IFN-γ, TNF, and MIP-1β responses for the indicated stimulations. In (**c**) and (**d**), bars represent median values. Paired *t*-test or Wilcoxon test were used for statistical analysis

We finally investigated whether NK cell function was modulated during the rapid clearance of HCV. Although observed changes were modest, certain traits were visible primarily when the patients were undergoing treatment (Fig. 8a, Supplementary Fig. 7a, b). Such changes included decreased ADCC responses as well as increased MIP-1β production in response to cytokine stimulation (Fig. 8a). However, these alterations appeared less connected to actual HCV clearance but rather attributable to the DAA treatment. Indeed, when the virus was completely eliminated and the patients were off treatment, NK cell function did not differ from that before initiation of treatment (Fig. 8a, b). In line with this observation, functional DEV and SDI remained stable throughout treatment (Fig. 8c, d). Additional experiments with the two long-term follow-up cohorts (Supplementary Table 2) substantiated these results with minor, if any, differences occurring during DAA treatment and only few alterations observed at weeks 60 (LDV/SOF) to 120 (SOF + RBV) after treatment (Supplementary Fig. 7a, b).

In summary, the observed imprint caused by chronic HCV on the NK cell compartment appears irreversible even after the rapid clearance of virus.

## Discussion

In the present study, we utilized high-dimensional flow cytometry technology combined with several novel metrics that capture immune population diversity and heterogeneity. This combination allowed us to determine the global impact of chronic HCV infection on the human NK cell compartment. Furthermore, taking advantage of recent developments within clinical medicine, the advent of highly effective DAAs against HCV^26^, we also investigated the durability of this imprint by comparing the NK cell compartment in the same patients before and after clearance of the chronic infection. Together, these technologies provide a comprehensive depiction of how chronic HCV infection alters human NK cell diversity.

Viruses that cause chronic infection exist in equilibrium with the host’s immune system. A wealth of studies in the human setting has shown how certain NK cell phenotypic and functional properties are affected by various chronic viral infections^21,22,30^. However, as we have learned, biology is enormously heterogeneous and rarely does a single parameter predict disease outcome. During the last years, the view of NK cells as phenotypically and functionally rather “unsophisticated” cells have been challenged. This scenario is underscored by a current study estimating that the NK cell compartment in a given individual consists of up to 30,000 phenotypically unique populations^6^. Such studies have become feasible because of technologic developments in multi-color flow cytometry and mass cytometry^31^. Previous work on NK cells in chronic HCV infection relied primarily on supervised conventional analysis of one marker at a time. Here we employed unsupervised SNE analysis^32,33^, allowing simultaneous assessment of thirteen NK cell receptors at the single-cell level. SNE has proven to be the most suitable dimensionality-reduction algorithm for high-dimensional flow and mass cytometry data^32,33^, and it offers the possibility of revealing formerly unrecognized cellular phenotypes or associations not available in low-dimensional space. Furthermore, since this analysis is unsupervised, the potential bias introduced by manual gating is circumvented.

The phenotype of NK cells in chronic HCV has previously been studied to some extent but with discrepancies among results published. For example, NKG2D expression in chronic HCV has been found to be increased^34^, unchanged^35–37^, or decreased^27^ in former reports. Some of these discrepancies might derive from different conventional flow cytometry analysis strategies. In our cohort, the expression of NKG2D clearly decreased on NK cells according to both SNE analysis and conventional single marker analysis. These discrepancies might in part be based on differing inclusion criteria or variations of subjects’ immune responses. Indeed, since the present patients with chronic HCV displayed elevated DEVs as compared to healthy controls, a plausible explanation is that chronic HCV infection, per se, does not drive one specific phenotype, but instead triggers patterns of phenotypic alterations that are unique for each patient. This becomes evident with regard to NKG2A expression, which is slightly reduced in the whole HCV patient cohort; however, some patients had expression levels that exceeded the median values for healthy controls. As a result, some individuals manifested a higher spread and elevated DEV.

The immune system, including the NK cell compartment, is a highly variable entity^6,38^. Whereas SNE takes measurements from multi-dimensional space and represents these values in two dimensions, which allows discrimination between more and less prevalent populations of phenotypically similar cells; this tool does not capture variation in diversity. Instead, for such assessments, we employed the two metrics of DEV and inverse SDI. DEV is an adjustment of cell-to-cell expression, for which an analysis framework has been published for T cells from healthy individuals^24^. DEV estimates inter-individual variation. We validated this metric on a large cohort of healthy donors stratified for CMV serostatus, where increased DEV was associated with markers known to be expressed by CMV-associated adaptive-like NK cell expansions. Therefore, we applied DEV to the thirteen NK cell receptors investigated here, as well as to the functional readouts, thereby showing that chronic HCV infection increases inter-individual variation within the NK cell compartment.

SDI is an established method within ecology, used to measure ecosystem diversity. In a recent report, SDI was used to document how NK cell diversity increases as individuals reach adulthood^25^. Adapting it to NK cell differentiation, we observed that NK cell SDI, as expected, decreased in CMV seropositive healthy individuals presumably as a result of CMV-driven expansions of distinct NK cell subsets. Furthermore, the intrahepatic NK cell compartment also presented with reduced SDI, reflective of the limited phenotypic diversity present within the tissue-resident CD56^bright^CD16^-^CD69^+^ NK cell subpopulation. Strikingly, our analysis revealed that chronic infection with HCV was also able to decrease SDI. This reduction of SDI implies that discrete subsets of NK cells are expanded in response to chronic HCV infection, a concept that is in line with the adaptive-like NK cells that appear in CMV seropositive individuals^14–16^. However, CMV was not driving the lower SDI in our chronic HCV cohorts, since stratification for CMV serostatus showed no significant difference. Taking into consideration that we observed no SDI reduction in acute HCV and patients with milder liver disease, we speculate that infections persisting for several years and possibly also advanced liver disease are able to influence the NK cell compartment towards lower repertoire diversity. What, then, is the implication of a lower SDI in chronic HCV? Given the fact that we could not detect a uniform adaptation of the NK cell differentiation pattern in these patients, we hypothesize that the chronic infection does not induce one specific phenotype, but rather allows enrichments/expansions of various subsets. Such variation in subpopulation frequencies might also be reflected in the increased DEV of patients with chronic HCV infection. Previously, increased NK cell diversity has been associated with risk of HIV acquisition in the acute infection setting^25,39^. Therefore, we speculate that, over time, chronic viral infections have different effects on the NK cell compartment than acute viral infections and that a reduced SDI could represent a disturbance in innate cellular immunity in line with memory inflation occurring within the CD8 T cell compartment in response to CMV^40^.

To assess factors leading to the imprint of chronic HCV infection on diversity of the NK cell receptor repertoire, we stratified for relevant clinical, virological, and genetic parameters in patients. This analysis revealed that the stage of liver disease influenced DEV, since cirrhotic patients presented with significantly higher DEVs. Furthermore, patients having *KIR2DL3* in combination with *HLA-C1/C1*, on the genetic level, had lower DEV than other patients. The mechanism for this is currently unclear. However, it is interesting to note that *KIR2DL3* together with *HLA-C1/C1* is beneficial for clearance of HCV^20^. Liver cirrhosis is known to affect the soluble inflammatory milieu during chronic HCV infection, thereby increasing plasma levels of adhesion molecules such as VCAM-1 and ICAM-1, as well as decreasing levels of the cytokines IL-17 and the growth factors FGF-β and PDGF-BB in plasma^41^. If so, alterations in the intrahepatic inflammatory milieu along with increased liver damage and inflammation, might affect the NK cell phenotype resulting in increased DEV. In contrast, however, we found only a weak link between SDI and degree of liver disease and no clear association between clinical parameters and results from the SNE analysis.

NK cell function has previously been shown to be altered during chronic HCV infection including reduced IFN-γ production capacity and elevated CD107a degranulation responses against target cells^34,36^. We could not detect such changes in the three cohorts analyzed in the current study. These contrasting results might be attributed to methodological differences where prior reports stimulated NK cells with IL-12 + IL-15^36^ or IL-12 + IL-21^34^ whereas we used IL-12 + IL-18. However, when performing a subgroup analysis comparing functional responses of patients with the lowest NK cell receptor repertoire diversity with others more similar to healthy controls, we noted that patients with receptor repertoire disturbances presented with a slight defect in cytokine production capacity. Finally, patients in previous studies were recruited in different clinical settings and there could always be confounders due to patient selection, comorbidities, co-medication, and environmental factors^36,42^. Thus, it is possible that grouping and selection of patients contribute to the contrasting results.

Lately, it has been shown that some HCV-induced alterations affecting the immune system undergo reinvigoration following clearance of the virus, as achieved with interferon-free DAA treatment^36,43,44^. In contrast to this, we and others demonstrated that peripheral blood MAIT cells were permanently lost with ensuing phenotypical alterations and dysfunction, despite rapid elimination of HCV^45,46^. In line with this, it has also been shown that chronic HCV infection induces an expansion of regulatory CD4^+^ T cells that also appears to persist long after DAA-mediated HCV clearance^47^. The present results suggest that the HCV-caused imprint on the NK cell compartment, with elevated DEV and reduced SDI, persists even after viral clearance up until two years after treatment cessation and viral clearance. However, some modulation of NK cell function took place during treatment, showing that NK cells, to a degree, are influenced by removal of the chronic infection.

Given that human NK cells have a turnover time in peripheral blood of approximately two weeks^48^, the entire NK cell compartment should have been renewed during the 120 weeks follow-up period when we studied the patients. However, it is still plausible that NK cell diversity is restored only at very-late time points after elimination of the virus. Indeed, within a subgroup of patients, we observed a reversal of the increased DEV in response to treatment. Since increased DEV was associated to degree of liver disease, and clearance of HCV leads to improvement of liver function in some patients, this might be the explanation behind the observed normalization of DEV. In contrast, we could show that only few phenotypic markers normalized over time, and that the main phenotypic alterations remained altered, both after conventional flow cytometry gating and SNE analysis. Elsewhere, both CMV infection and exposure to cytokines caused epigenetic modifications within NK cell populations leading to long-lasting effects on the NK cell phenotype and function^49–51^. Thus, chronic HCV infection conceivably induces similar epigenetic alterations underlying the stably reduced SDI observed here long after the virus had been cleared.

In summary, we have used high-dimensional single-cell analysis to determine the effect of a chronic viral infection on diversity in the NK cell repertoire of humans. The results indicate that chronic HCV significantly impacts human NK cells. This effect was long lasting since it persisted after clearance of the virus and could, at least partially, be explained by the degree of liver disease. Our results provide a novel framework for moving future studies beyond single-parameter analysis. To be able to more completely grasp the vast complexity of the human NK cell compartment could aid in delineating the implications of these cells in settings of human disease.

## Methods

### Healthy control and patient cohorts

Three cohorts of patients with chronic HCV infection were studied (with corresponding cohorts of healthy controls), all recruited at the  Department of Gastroenterology, Hepatology, and Endocrinology at Hannover Medical School in Germany. The first cohort consisted of twenty-six patients with chronic HCV infection, as well as ten community-matched healthy controls (Fig. 1). These patients were studied longitudinally before, during, and after 24 weeks of IFN-free treatment with SOF + RBV. PBMC samples were taken before treatment, at weeks 4, 12, and 24 during treatment, as well as 36 or 48 weeks after treatment onset (12 and 24 weeks, respectively, after the end of therapy) (Fig. 1). Eight out of these 26 patients experienced a viral relapse after the end of therapy. The second cohort consisted of nine chronic HCV patients also treated with SOF + RBV with long-term follow-up samples. In this second cohort, on treatment samples were taken at weeks 12 or 24 and follow-up at week 120 (week 96 after end of treatment). Six of the patients were part of the original cohort described above but now with additional very-long-term follow-up samples (when analysis on combined cohorts are performed, these patients are censored). For six of these patients, no baseline sample was available, but instead those samples taken early during treatment were used (five patients at week 1 and one patient at week 4). The third cohort consisted of 12 patients receiving LDV/SOF treatment. Samples were taken at baseline, at weeks 2, 4, 8, or 12 during therapy, and at weeks 36, 60, and/or 108 (equaling follow-up weeks 24, 48 and 96). Additionally, 12 patients with acute HCV, from the HepNet-aHCV-IV study (EudraCT # 2013-001081-42, clinicaltrial.gov # NCT02309918), were analyzed^52^. All HCV patients were HIV-, HBV-, and HDV-negative. For validation of SDI and DEV, 202 healthy blood donors were studied^16^. Moreover, nine liver samples, obtained as previously described from unaffected tissue removed during surgery for colorectal liver metastasis^29^, were included to assess DEV and SDI for intrahepatic NK cells. The ethics committee of Hannover Medical School (Study numbers 2148–2014 and 6751 M) and the Regional Ethics Committee in Stockholm, Sweden (Study number 2013/2285-31) approved the study. All patients provided oral and written informed consent. Supplementary Table 1 and 2 summarize the clinical characteristics of the chronic HCV patients and healthy controls. Clinical data regarding HCV and CMV status, stage of liver fibrosis and cirrhosis, and liver inflammation were available from routine clinical diagnostics, as previously described in depth for the first cohort^53^.

### *KIR* and *KIR*-ligand genotyping

DNA from all patients was extracted from thawed PBMC with the DNeasy Blood & Tissue Kit (Qiagen, catalog number: 69504) according to the manufacturer’s instructions. *KIR* genotyping was performed with the *KIR* SSO Genotyping Test (One Lambda, catalog number: RSSOKIR) and *KIR*-ligands were identified with the *KIR HLA* Ligand SSP typing kit (Olerup, catalog number: 104.201-12).

### Flow cytometry

Flow cytometry staining was performed as previously described (Figs. 1 and 2a)^54^. In brief, cryopreserved PBMCs were thawed in complete medium (RPMI supplemented with 10% FCS and 2 mM L-glutamine), washed twice, and resuspended in PBS containing 2% FCS and 2 mM EDTA. All flow cytometry stainings were performed at room temperature in the dark. Supplementary Table 4 contains a list of antibodies used. PBMCs were stained immediately after thawing for 20 min with the desired mixture of antibodies. Subsequently, the cells were washed twice and then stained for 15 min with streptavidin conjugates. After washing twice, the cells were fixed for 30 min at RT with eBioscience Fixative (FOXP3/Transcription Buffer staining set, eBioscience) and acquired on a LSR Fortessa cell analyzer (BD Biosciences) equipped with 355 nm, 405 nm, 488 nm, 561 nm, and 640 nm lasers. Flow cytometry data were analyzed with FlowJo software V.9.8 (Treestar Inc., Ashland, OR). Staining panels are listed in Supplementary Table 5.

### Functional NK cell assays and analysis

To assess the functional capacity of NK cells, four different stimuli were applied. As a control, PBMCs were thawed and rested for 24 h in medium. To assess cytotoxicity, K562 cells were added at a 10:1 effector to target ratio for the last 6 h; to assess cytokine responses, IL-12 (10 ng/mL) and IL-18 (100 ng/mL) were added for 18 h; and for measuring ADCC, 721.221 cells were added at a 10:1 effector to target ratio together with 1μg/mL Rituximab for the last 6 h. CD107a-FITC was present throughout the assay to assess degranulation. Brefeldin A and Monensin were added for the last 5 h of stimulation. After incubation, staining for flow cytometry was performed as described above. After surface staining, fixation, and permeabilization, intracellular-staining antibodies were added for 30 min at RT and then washed twice with washing buffer. For analysis of multifunctional responses, SPICE version 5.3 (National Institutes of Health, Bethesda, MD)^55^ was used. A Boolean gate of NK cells positive for MIP-1β, CD107a, TNF, and IFN-γ was used for assessing multifunctionality.

### Stochastic neighbor embedding analysis

SNE analysis was employed as a dimensionality reductive method to cluster and visualize the phenotypes of NK cells in HCV patients and healthy controls as previously described^33^. Briefly, data from 2000 CD56^dim^ or 1000 CD56^bright^ NK cells were exported for each control and HCV patient sample. These were electronically barcoded for grouping and retrospective identification of individual samples. Samples with few events in the respective subset were excluded from SNE analysis, as well as samples from two HCV patients and two healthy controls due to differing staining panels. For comparison of longitudinal samples, only data from patients with the respectively available time points were included. Finally, the data were analyzed using Barnes-Hut SNE in R (version 3.3.1) as previously described^33^.

### Metrics of immune system composition

Expression of NKG2A, CD57, panKIR, CD38, CD25, Granzyme B, NKG2D, Ki-67, NKp30, NKp46, DNAM, CD69, PD-1, and CD161 on both CD56^dim^ and CD56^brigh^ NK cells was included in the measurement of DEV. DEV was calculated with the following formula:

1 $${\mathrm{DEV}} = \sqrt {\frac{1}{{N - 1}} \ast {\sum} {\left( {\frac{{y_i}}{{\bar x}} - y_m} \right) ^{{\wedge}} 2} }$$

where *N* is the size of group *y*,$y_i$ is the individual expression level of a given donor in group *y*, $\bar x$ is the median expression level of all samples analyzed, and $y_m$ is the mean of group $\frac{{y_i}}{{\bar x}}$ for the samples contained in group *y*. In brief, values for each parameter were normalized to the median of all samples. The standard deviation was then calculated for the respective cohort. This measurement determines the variation within the respective populations without losing the relationship to the level of marker expression. To calculate DEV of the functional readouts, the standard deviation of the median-normalized values for the parameters that gave positive responses in the respective treatment were used thus: for all conditions CD107a, MIP-1β, IFN-γ, TNF, and GM-CSF, for 721.221 and Rituximab stimulation additionally HLA-DR, and for cytokine priming additionally HLA-DR, CD25 and CD69 expression.

The inverse SDI was calculated as previously described^6,39^:

2 $${\mathrm{SDI = }}\frac{1}{{\mathop {\sum }\nolimits_{{\it{i}} = 1}^S p_i^2}}$$

where *S* is the amount of all species in the population and _*pi*_ the proportion of the *i’*th species. For phenotypic SDI, the Boolean gate for the NK cell differentiation markers CD56^bright^, CD57, NKG2A, and panKIR was applied. For CD56^bright^ NK cells, only NKG2A^+^ KIR^+^ or KIR^−^ populations were considered. Functional SDI was assessed with the Boolean gate for multifunctional analysis of CD107a, MIP-1β, IFN-γ, and TNF.

### Statistical analysis

GraphPad Prism version 6.0b was applied for data analyses. Normal distribution of data was evaluated using the D’Agostino & Pearson omnibus normality test. For normally distributed data, analyses were performed using the unpaired *t*-test or paired *t*-test based on the data sets that were compared with each other. For non-normally distributed data, the Mann–Whitney test or Wilcoxon matched-pairs signed rank test was used. To test for equal distribution of clinical parameters, Fisher’s exact test was performed with the respective other parameter. Unless otherwise stated, n.s. stands for not significant, *p < 0.05, **p < 0.01, and ***p < 0.001.

### Data availability

The authors declare that the data supporting the findings of this study are available within the article and its supplementary information files, or are available upon reasonable requests to the authors.

## Electronic supplementary material

Supplementary Information
