# Supplementary material for: Chronic hepatitis C virus infection irreversibly impacts human natural killer cell repertoire diversity
Source: Nat Commun. 2018 Jun 11;9:2275. doi: 10.1038/s41467-018-04685-9 (PMC5995831; doi:10.1038/s41467-018-04685-9)
Supplement: Supplementary file 1 — Supplementary Information [file 41467_2018_4685_MOESM1_ESM.pdf]

Supplementary Information

Chronic hepatitis C virus infection irreversibly impacts human NK cell repertoire diversity

Benedikt Strunz, Julia Hengst, Katja Deterding, Michael P. Manns, Markus Cornberg, Hans-Gustaf Ljunggren, Heiner Wedemeyer, and Niklas K. Björkström

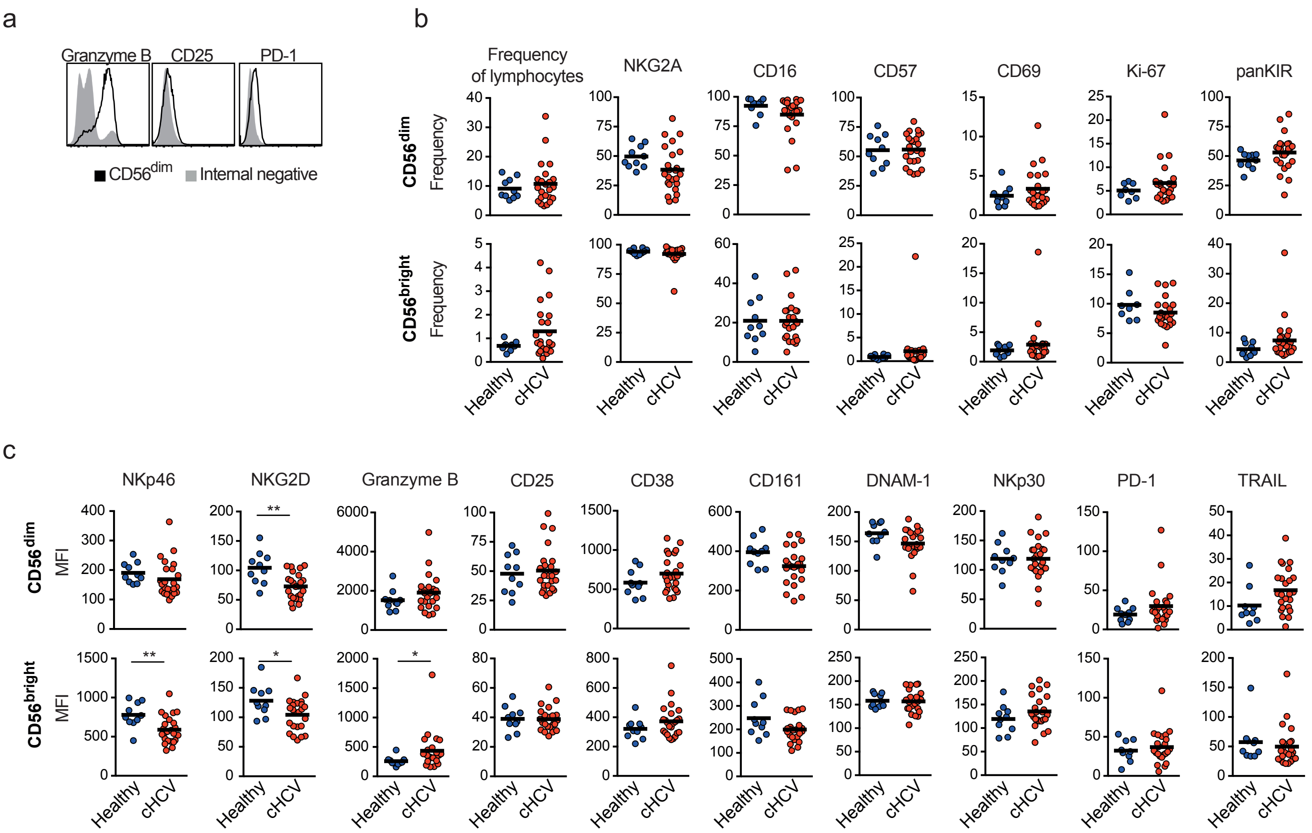

Supplementary Figure 1. Conventional flow cytometry analysis of NK cell-associated markers. (**a**) Histograms from representative stainings for markers not included in SNE analysis. (**b**) Summarized frequencies of indicated receptors expressed within CD56^{dim} and CD56^{bright} NK cells from healthy controls (*n*=10) and patients with chronic HCV (*n*=24). (**c**) Summarized expression levels (mean fluorescence intensity, MFI) of the indicated receptors within CD56^{dim} and CD56^{bright} NK cells from healthy controls (*n*=10) and patients with chronic HCV (*n*=24). In **b** and **c**, bars represent mean values, * $p<0.05$, ** $p<0.01$. Mann-Whitney or unpaired t-test were used for statistical analysis.

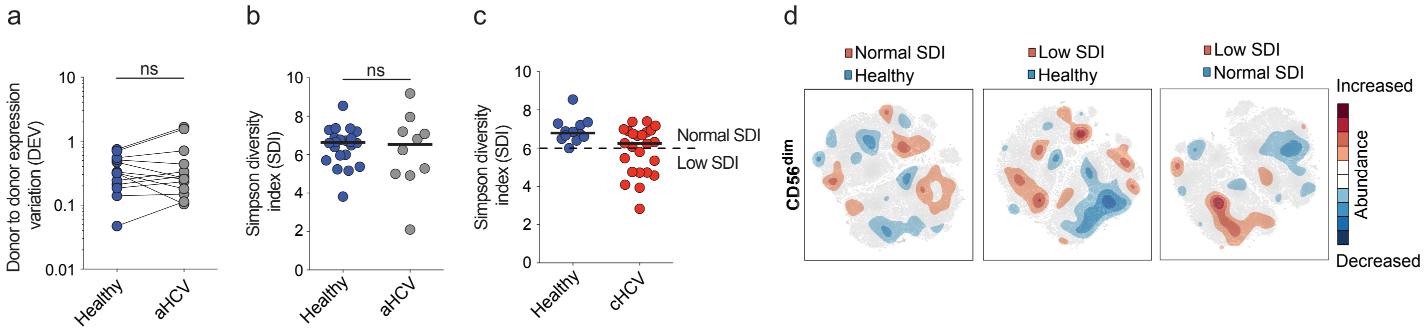

Supplementary Figure 2. Modulation of the inverse Simpson diversity index (SDI) and donor-to-donor expression variation (DEV) of NK cells. (**a**) DEV for healthy controls (*n*=5) and acute HCV patients (*n*=5) and (**b**) SDI for healthy individuals (*n*=22) and acute HCV patients (*n*=10). (**c**) Chronic HCV patients were grouped in normal (SDI values above the lowest SDI for the healthy controls, *n*=13) and low SDI (*n*=8). Based on this grouping, **(d)** stochastic neighbor embedding analysis was performed comparing healthy controls (*n*=8) with chronic HCV patients (“low” (*n*=8) and “normal” SDI (*n*=13)) and also the two chronic HCV cohorts.

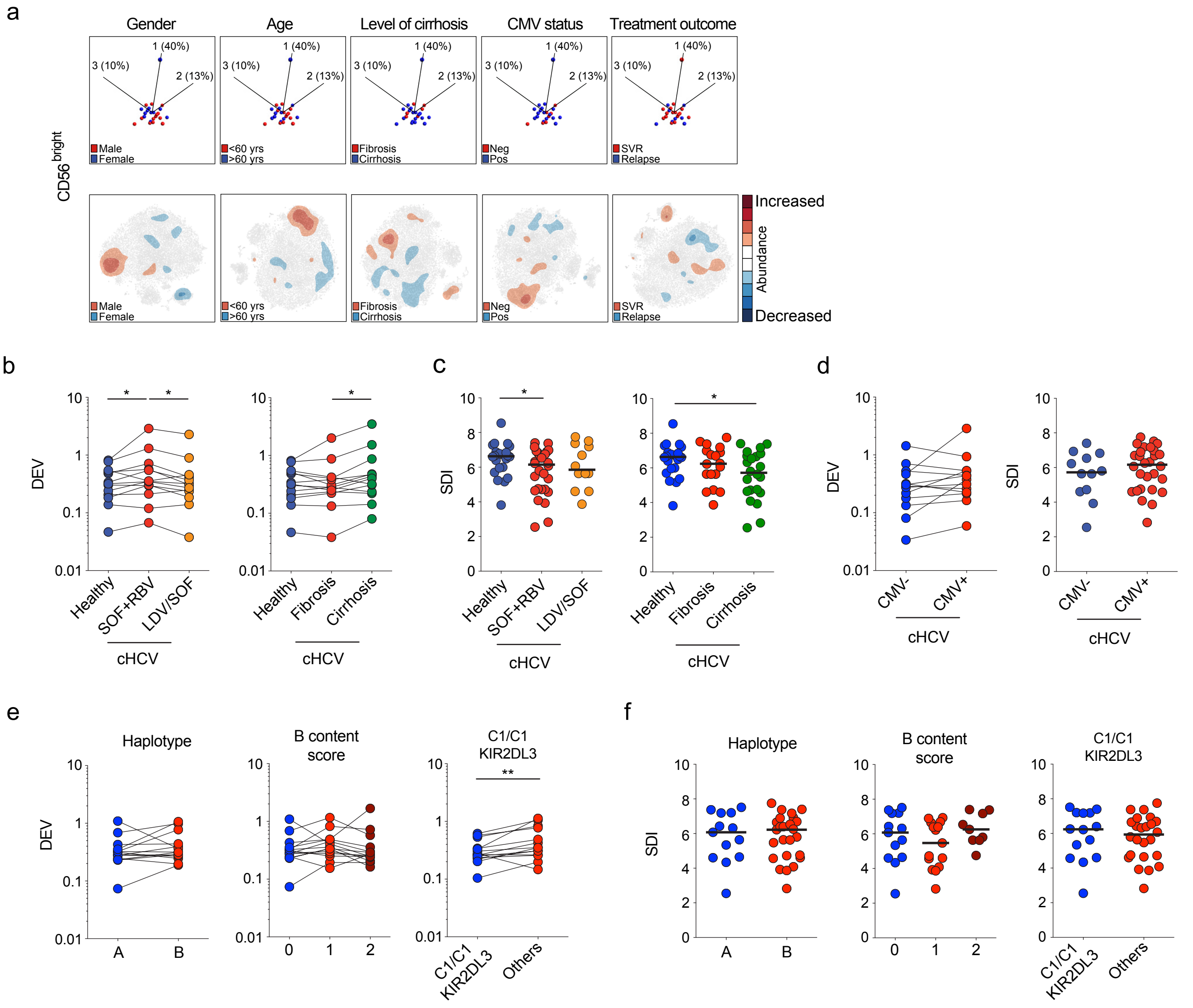

Supplementary Figure 3. Influence of clinical parameters on donor-to-donor expression variation (DEV) and inverse Simpson diversity index (SDI) in chronic HCV patients. (**a**) Principal component analysis (PCA) (upper row) and stochastic neighbor embedding (SNE) analysis (lower row) of CD56^{bright} NK cells in relation to clinical parameters. Included factors were gender (male *n*=13 and female *n*=11); age, <60 years (*n*=13), >60 years (*n*=11); cirrhosis-status defined as Fibroscan value >14.5 kPa, fibrosis (*n*=6), cirrhosis (*n*=18); CMV status, seronegative (*n*=7), seropositive (*n*=17); treatment outcome, sustained virological response (SVR) (*n*=16), relapse (*n*=8). One patient who was excluded from SNE analysis represents a significant outlier (data not shown). (**b**) DEV for different chronic HCV cohorts (DEV calculations were based on healthy *n*=13, sofosbuvir+ribavirin (SOF+RBV) *n*=9, ledipasvir/sofosbuvir (LDV/SOF) *n*=10 in left plot; right plot, healthy *n*=13, chronic HCV patients grouped by Fibroscan values into fibrotic *n*=13 and cirrhotic *n*=6).

(**c**) SDI for different chronic HCV cohorts (left plot, healthy *n*=22, SOF+RBV *n*=28, LDV/SOF *n*=12; right panel, healthy *n*=22, chronic HCV patients grouped into fibrotic *n*=18 and cirrhotic *n*=22). (**d**) DEV and SDI in relation to CMV serostatus. DEV data representative of analyses from two chronic HCV-cohorts with long-term follow-up data (*n*=5-14) and SDI data representative of the entire cohort with CMV seronegative (*n*=12) and CMV seropositive (*n*=28) individuals. (**e-f**) DEV and SDI for chronic HCV patients depending on *KIR/KIR*-ligand genotypes (see Supplementary Table 3). (**e**) Left plot summarizes *n*=6-18, middle plot *n*=6-12, and right plot *n*=9-15. (**f**) Left plot shows *n*=13-25, middle plot *n*=9-15, and right plot *n*=14-24. Bars represent median values, * indicates $p<0.05$, ** = $p<0.01$. Wilcoxon test or unpaired t-test were used for statistical analysis.

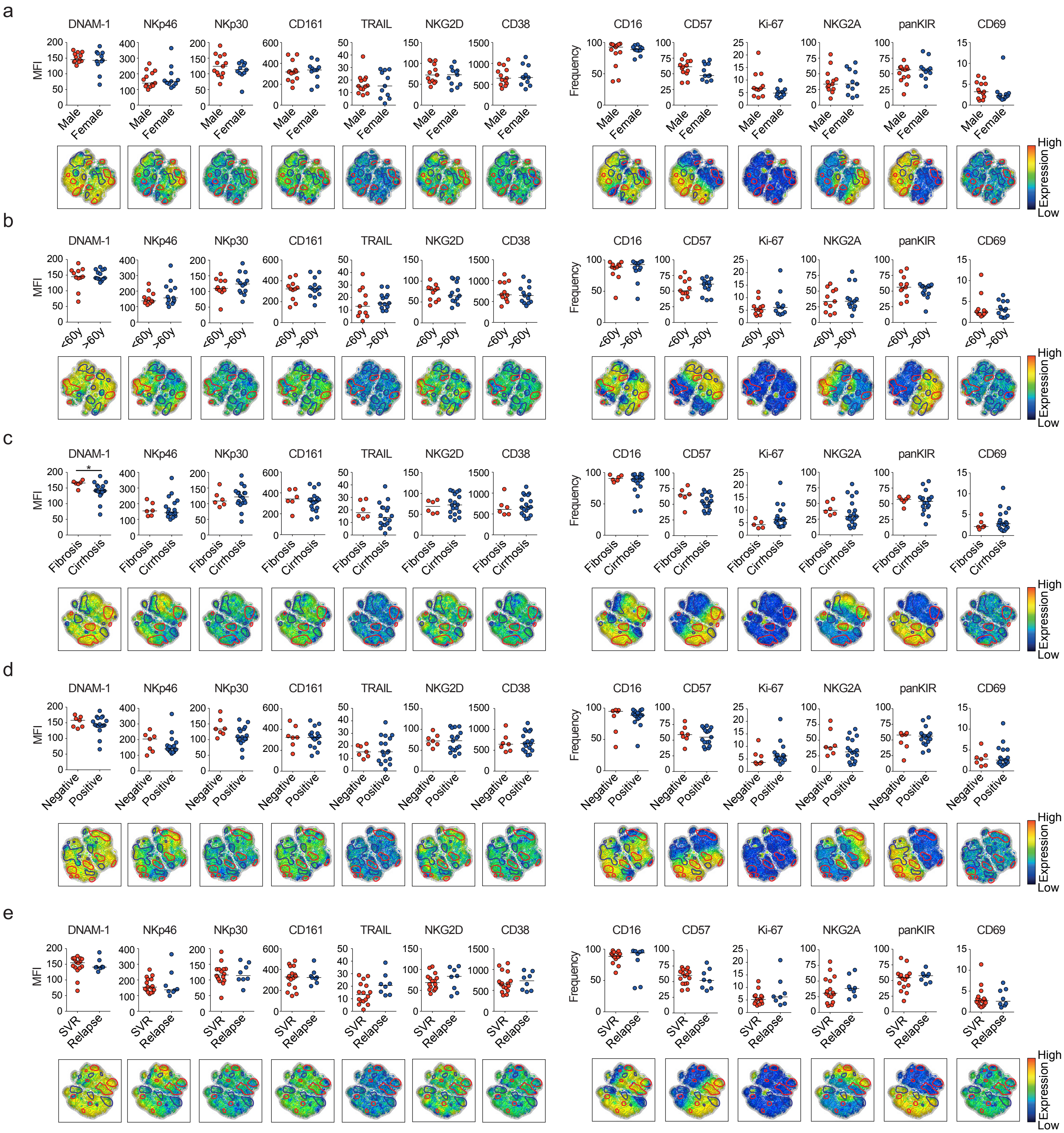

Supplementary Figure 4. Single parameter analysis stratified for clinical parameters. Results of single parameter analysis in conventional gating (**a-e**, upper row) and in the respective stochastic neighbor embedding analysis (**a-e**, lower row) for CD56^{dim} NK cells. Data were grouped based on (**a**) gender, (**b**) age, (**c**) cirrhosis status, (**d**) CMV serostatus, and (**e**) treatment outcome following therapy with direct-acting antivirals (DAA). Bars represent median values, * p <0.05. Mann-Whitney test was used for statistical analysis.

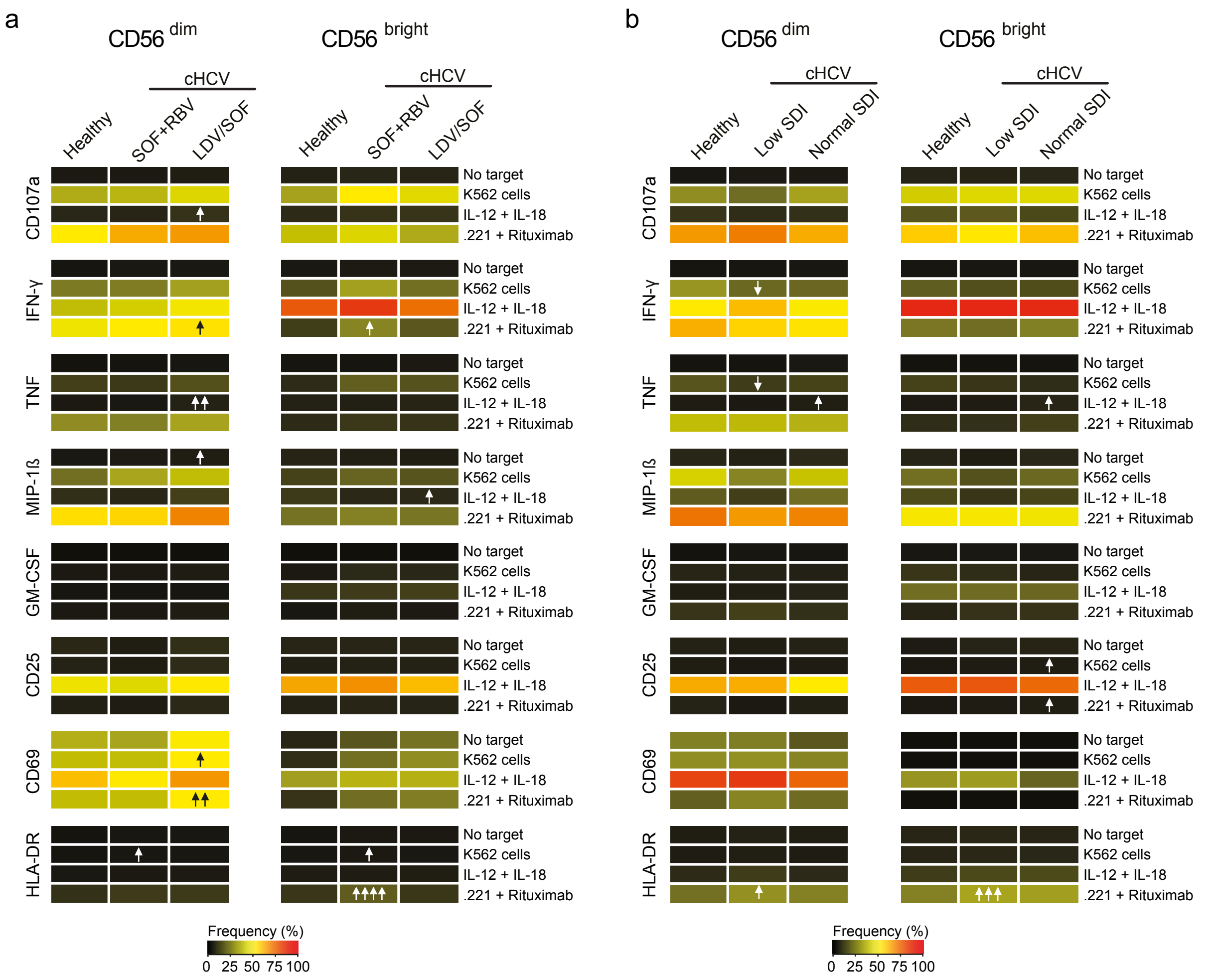

Supplementary Figure 5. Functional analysis of NK cells at baseline in chronic HCV patients treated with different direct-acting antiviral (DAA) treatment regiments. (a) Heatmaps summarizing the median frequency of responding cells or median value of the mean fluorescence intensity (MFI) (CD69 and MIP-1β) for the indicated functional readouts after stimulation within CD56^{dim} and CD56^{bright} NK cells for healthy controls (*n*=11), sofosbuvir+ribavirin (SOF+RBV) treated chronic HCV patients (*n*=9), ledipasvir/sofosbuvir (LDV/SOF) treated chronic HCV patients (*n*=10). (**b**) Heatmaps summarizing the median frequency of responding cells or median MFI (CD69 and MIP-1β) for the indicated functional readouts after stimulation within CD56^{dim} and CD56^{bright} NK cells for healthy controls and chronic HCV patients with "low" (*n*=9) or "normal" (*n*=12) inverse Simpson diversity index (SDI) (see Supplementary Fig. 2). Number of arrows, irrespective of color indicate the level of significance (from ↑ = $p<0.05$ to ↑↑↑↑= $p<0.0001$) compared to healthy controls and the direction reflects increased or decreased expression. Mann-Whitney or unpaired t-test were used for statistical analysis.

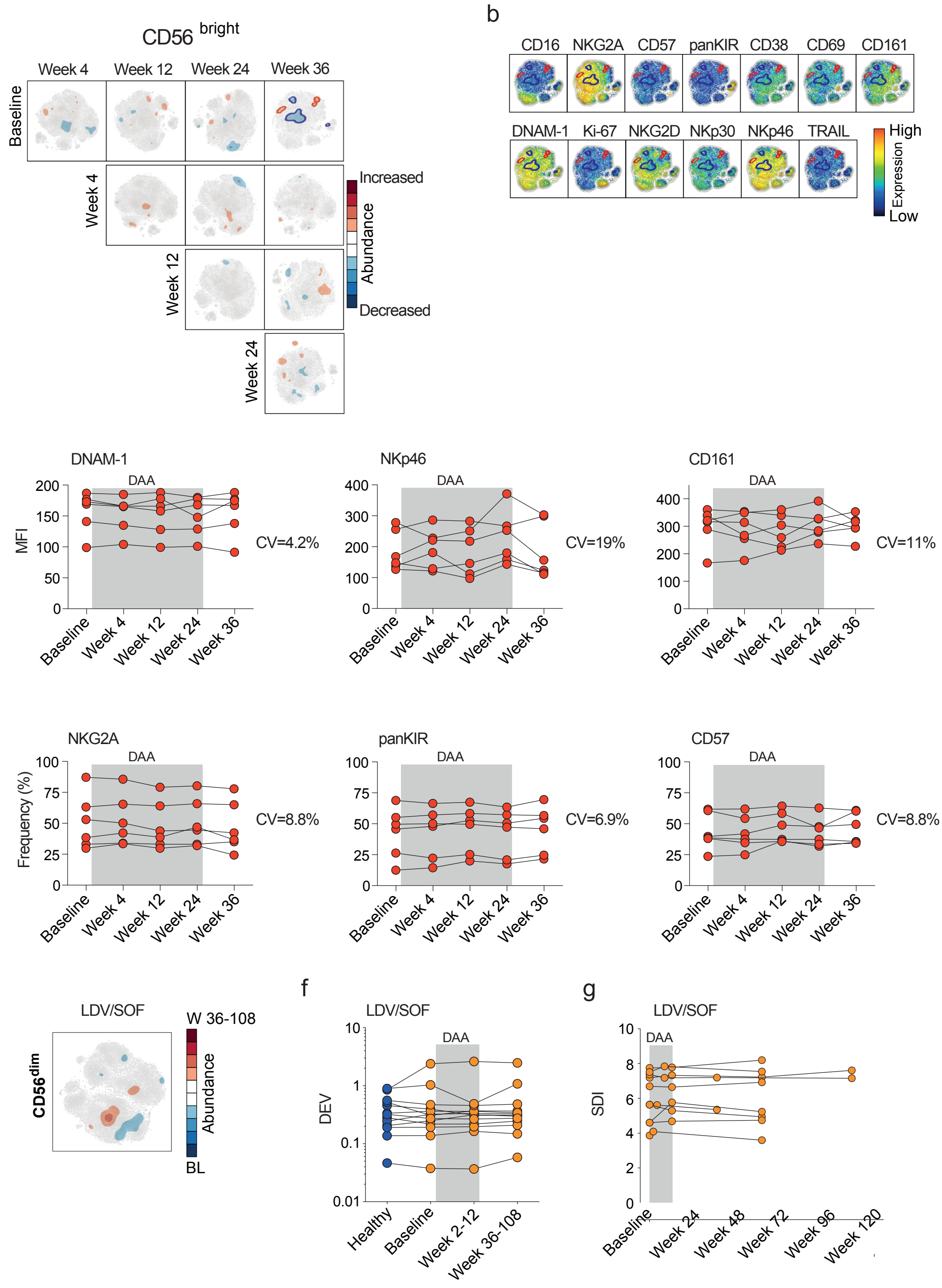

Supplementary Figure 6. Phenotypic analysis of NK cells during and after successful therapy with direct-acting antivirals (DAA). (**a**) Pairwise residual plots from stochastic neighbor embedding (SNE) analysis comparing the indicated time-points for CD56^{bright} NK cells from patients with chronic HCV (*n*=19-19-16-11 for the comparison baseline to weeks 4-12-24-36, *n*=19-16-11 for week 4 to weeks 12-24-36, *n*=16-11 for week 12 to weeks 24-35 and *n*=10 for week 24 to week 36). (**b**) The residual plot comparing baseline with week 36 from (**a**) is projected onto individual density plots for the 13 markers included in the SNE analysis.

(**c**) Representative data of five sofosbuvir+ribavirin (SOF+RBV) patients for expression levels of DNAM-1, NKp46 and CD161 and (**d**) their expression of NKG2A, panKIR, and CD57 on total NK cells over time. CV indicates the median coefficient of variation from all patients and was calculated for each patient over time. (**e**) Residual plot from SNE analysis when comparing baseline to long-term follow up within the ledipasvir/sofosbuvir (LDV/SOF) cohort. (**f**) Donor-to-donor expression variation (DEV) over time in chronic HCV patients treated with LDV/SOF (*n*=13 for healthy controls, *n*=10 for HCV patients). (**g**) Inverse Simpson diversity index (SDI) over time in chronic HCV patients treated with LDV/SOF (*n*=10).

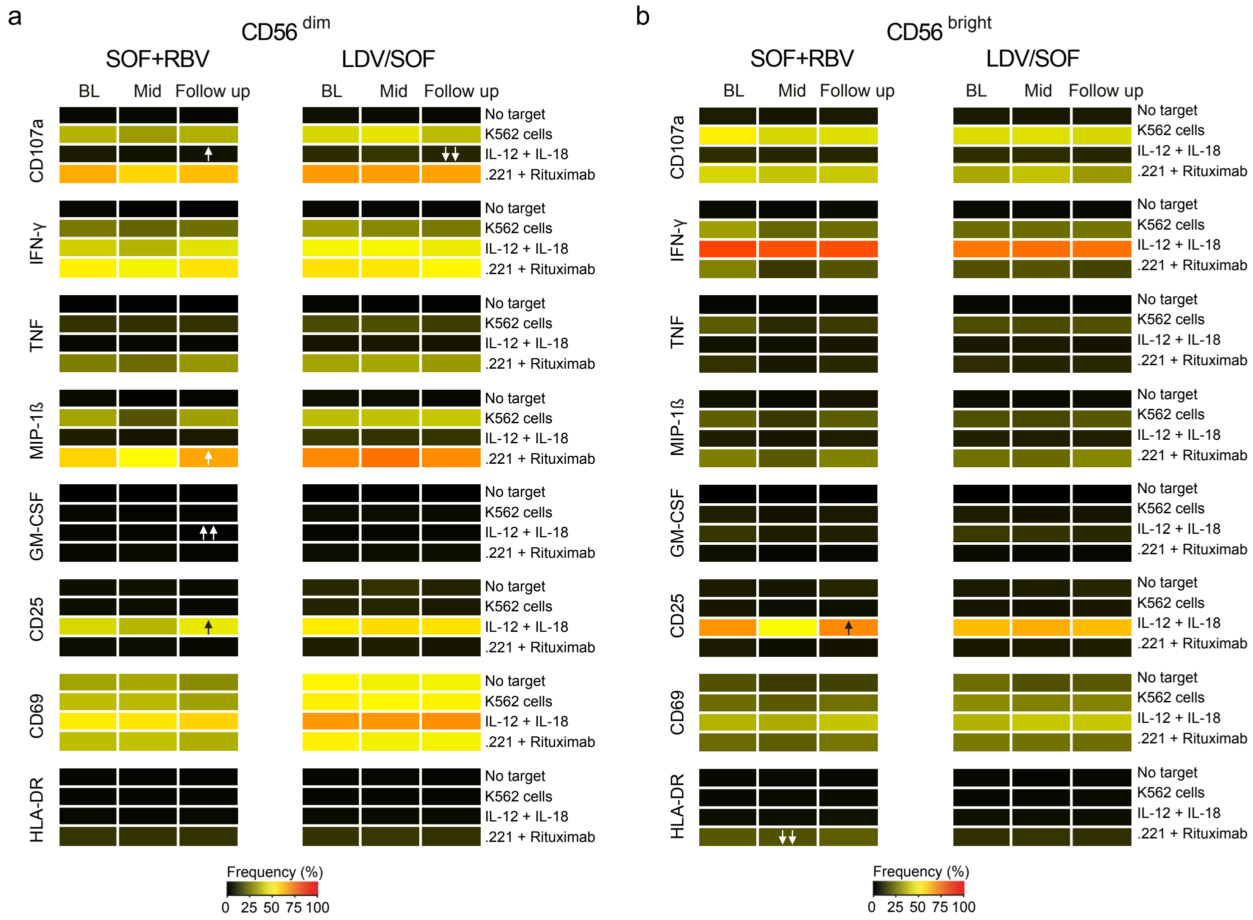

Supplementary Figure 7. Modulation of NK cell function during and after clearance of HCV. (**a**) Heatmaps summarizing the median frequency of responding cells or median value of the mean fluorescence intensity (MFI) (CD69 and MIP-1β) for the indicated functional readouts after stimulation within CD56^{dim} NK cells for chronic HCV patients receiving sofosbuvir+ribavirin (SOF+RBV) or ledipasvir/sofosbuvir (LDV/SOF) assessed over time. (**b**) Heatmaps summarizing the median frequency of responding cells or median MFI (CD69 and MIP-1β) for the indicated functional readouts after stimulation within CD56^{bright} NK cells for chronic HCV patients receiving SOF+RBV or LDV/SOF assessed over time. Black and white arrows indicate significant differences compared to baseline (🡅=$p<0.05$, 🡅🡅=$p<0.01$). Wilcoxon or paired t-test were used for statistical analysis.

Supplementary Table 1. Clinical characteristics of the initial chronic HCV patient cohort

	**Healthy controls**	**HCV patients (SOF+RBV)**
n (m/f)	10 (4/6)	26 (14/12)
Age	32 (26-63)	58 (41-73)
HCV genotype (1/2/3/4)	NA	11/1/13/1
Outcome (SVR/Relapse)	NA	18/8
HCV RNA (IU/mL)	NA	$7.25*10^5$ ($1.6*10^3$-$7.6*10^6$)
ALT (U/l)	NA	81 (31-415)
AST (U/l)	NA	79 (31-223)
γGT (U/l)	NA	88 (21-420)
Absolute lymphocyte count	NA	1,450 (100-3,700)
FibroScan (kPa)	NA	21.0 (5.8-48.0)

Supplementary Table 2. Clinical characteristics of validation and long-term follow-up chronic HCV patient cohorts

	**Healthy controls**	**HCV patients (SOF+RBV)**	**HCV patients (LDV/RBV)**
n (m/f)	13 (3/10)	9 (2/7)	12(6/6)
Age	43 (26-59)	60 (50-74)	54 (25-72)
HCV genotype (1/2/3/4)		5/1/2/1	12/0/0/0
outcome (SVR/Relapse)		9/0	12
HCV RNA (IU/mL)		$6.8*10^5$ ($1.6*10^3$-$1.1*10^7$)	$4.95*10^5$ ($5*10^1$-$6.3*10^6$)
ALT (U/l)		67 (32-132)	55.5 (18-90)
AST (U/l)		58 (40-98)	38 (24-61)
gGT (U/l)		82 (21-280)	35.5 (16-137)
Absolute lymphocyte count		1,400 (800-2,500)	1,900 (1,100-3,300)
FibroScan (kPa)		16.6 (8.1-25.1)	4.65 (2.2-7.1)

All parameters are summarized as median values and range.
NA= Not applicable
ALT = Alanine aminotransferase
AST= Aspartat aminotransferase
γGT= Gamma glutamyltransferase

Supplementary Table 3. KIR and HLA genotyping of all HCV patients.

#	Therapy	HLA-C	Haplotype	Locus	C1C1 +2DL3	B content score	Treatment outcome
1	SOF+RBV	C1/C2	A	CenA/CenA	no	0	SVR
2	SOF+RBV	C1/C2	A	CenA/CenA	no	0	SVR
3	SOF+RBV	C1/C1	A	CenA/CenA	yes	0	SVR
4	SOF+RBV	C1/C1	A	CenA/CenA	yes	0	Rel
5	SOF+RBV	C1/C1	A	CenA/CenA	yes	0	SVR
6	SOF+RBV	C1/C2	A	CenA/CenA	no	0	Rel
7	SOF+RBV	C1/C1	A	CenA/CenA	yes	0	SVR
8	SOF+RBV	C1/C1	A	CenA/CenA	yes	0	SVR
9	SOF+RBV	C1/C2	B	CenA/TelB	no	1	SVR
10	SOF+RBV	C2/C2	B	CenB/TelA	no	1	Rel
11	SOF+RBV	C1/C2	B	CenB/TelA	no	1	SVR
12	SOF+RBV	C1/C2	B	CenB/TelA	no	1	SVR
13	SOF+RBV	C1/C2	B	CenB/TelA	no	1	SVR
14	SOF+RBV	C1/C2	B	CenA/TelB	no	1	Rel
15	SOF+RBV	C1/C2	B	CenA/TelB	no	1	SVR
16	SOF+RBV	C1/C2	B	CenA/TelB	no	1	SVR
17	SOF+RBV	C1/C1	B	CenA/TelB	yes	1	Rel
18	SOF+RBV	C1/C1	B	CenB/TelA	yes	1	SVR
19	SOF+RBV	C1/C1	B	CenB/TelA	yes	1	SVR
20	SOF+RBV	C1/C2	B	CenA/TelB	no	1	Rel
21	SOF+RBV	C1/C1	B	CenB/TelB	yes	2	Rel
22	SOF+RBV	C2/C2	B	CenB/TelB	no	2	Rel
23	SOF+RBV	C1/C1	B	CenB/TelB	yes	2	SVR
24	SOF+RBV	C1/C1	B	CenB/TelB	yes	2	SVR
25	SOF+RBV	C2/C2	B	CenB/TelA	no	2	SVR

26	SOF+RBV	C1/C2	B	CenB/TelB	no	2	SVR
27	SOF+RBV	C1/C2	A	CenA/CenA	no	0	SVR
28	SOF+RBV	C1/C1	B	CenB/TelA	no	2	SVR
29	SOF+RBV	C1/C1	B	CenB/TelB	yes	2	SVR
30	LDV/SOF	C1/C1	A	CenA/CenA	yes	0	SVR
31	LDV/SOF	C2/C2	A	CenA/CenA	no	0	SVR
32	LDV/SOF	C1/C2	A	CenA/CenA	no	0	SVR
33	LDV/SOF	C1/C1	A	CenA/CenA	yes	0	SVR
34	LDV/SOF	C2/C2	A	CenA/CenA	no	0	SVR
35	LDV/SOF	C1/C1	B	CenA/TelB	yes	1	SVR
36	LDV/SOF	C1/C2	B	CenB/TelA	no	1	SVR
37	LDV/SOF	C1/C2	B	CenB/TelA	no	1	SVR
38	LDV/SOF	C1/C2	B	CenB/TelB	no	2	SVR
39	LDV/SOF	C1/C2	B	CenB/TelB	no	3	SVR

Supplementary Table 4. Antibodies for flow cytometry.

Antigen	Fluorochrome	Clone	Dilution	Company
CD3	PE-Cy5	UCHT1	1/100	Beckman Coulter
CD4	PE-Cy5	OKT4	1/200	BioLegend
CD4	BB515	RPA-T4	1/100	BD Biosciences
CD14	V500	M5E2	1/100	BD Biosciences
CD16	AF700	3G8	1/400	BD Biosciences
CD16	BV786	3G8	1/100	BD Biosciences
CD19	BV510	SJ25C1	1/100	BD Biosciences
CD25	Biotin	BC96	1/100	BioLegend
CD25	BV711	BC96	1/50	BioLegend
CD38	BV711	HIT2	1/50	BD Biosciences
CD56	ECD	N901	1/50	Beckman Coulter
CD56	PE-CF594	NCAM16.2	1/100	BD Biosciences
CD56	PE-Cy7	NCAM16.2	1/50	BD Biosciences
CD57	Purified	TB01	1/00	eBioscience
CD57	BV605	NK-1	1/200	BD Biosciences
CD69	APC-Cy7	FN50	1/100	BD Biosciences
CD107a	FITC	H4A3	1/100	BD Biosciences
CD107a	VioBright FITC	H4A3	1/100	Miltenyi Biotec
CD161	BV605	HP-3G10	1/17	BioLegend
DNAM-1	FITC	DX11	1/25	BD Biosciences
DNAM-1	BB515	DX11	1/25	BD Biosciences
GM-CSF	PE-CF594	BVD2-21C11	1/100	BD Biosciences
Granzyme B	AF700	GB11	1/200	BD Biosciences
HLA-DR	BV785	L243	1/50	BioLegend
IFNγ	BV421	B27	1/200	BD Biosciences
Ki-67	AF700	B56	1/100	BD Biosciences
Ki-67	BUV395	B56	1/100	BD Biosciences

KIR2DL1/S1	PE-Cy5.5	EB6B	1/17	Beckman Coulter
KIR2DL2/L3/S2	PE-Cy5.5	GL183	1/50	Beckman Coulter
NKG2A	APC	Z199	1/25	Beckman Coulter
NKG2D	PE-Cy7	1D11	1/100	BioLegend
NKp30	Biotin	P30-15	1/100	BioLegend
NKp46	BV421	9E2	1/50	BD Biosciences
MIP-1β	PE	D21-1351	1/50	BD Biosciences
PD-1	PE	EH12.1	1/50	BD Biosciences
PD-1	BUV737	EH12.1	1/20	BD Biosciences
TNF	BV650	Mab11	1/25	BD Biosciences
TRAIL	PE	RIK-2	1/25	BD Biosciences
	Anti-mouse IgM BV650	R6-60.2	1/50	BD Biosciences
	Streptavidin Qdot 585	NA	1/200	ThermoFisher
	LIVE/DEAD Fixable Aqua Dead Cell Stain Kit	NA	1/100	ThermoFisher
	Foxp3 Transcription Factor Staining Buffer Kit	NA		ThermoFisher

Supplementary Table 5. Flow cytometry staining panel.

Channel	Panel 1	Panel 2	Panel 3	Panel 4
FITC / BB515	DNAM-1	CD4	CD107a	DNAM-1
APC	NKG2A		NKG2A	NKG2A
AF700	Ki-67	Granzyme B	CD16	Ki-67
APC-Cy7	CD69		CD69	CD69
BV421	NKp46		IFNγ	NKp46
V500 / BV510	CD14	CD14	CD14	CD14
	CD19	CD19	CD19	CD19
	DCM	DCM	DCM	DCM
BV570	NKp30 biotin + Streptavidin Qdot 585		CD25	NKp30 biotin + Streptavidin Qdot 585
BV605	CD161		CD57	CD161
BV650	CD57 + anti-IgM	CD57 + anti-IgM	TNF	CD57 + anti-IgM
BV711	CD38		CD38	CD38
BV785	CD16		HLA-DR	CD16
PE	TRAIL	PD-1	MIP-1β	TRAIL
PE-CF594 / ECD	CD56		GM-CSF	CD56
PE-Cy5	CD3	CD3	CD3	CD3
	CD4		CD4	CD4
PE-Cy5.5	KIR2DL1/S1		KIR2DL1/S1	KIR2DL1/S1
	KIR2DL2/L3/S2		KIR2DL2/L3/S2	KIR2DL2/L3/S2

PE-Cy7	NKG2D		CD56	NKG2D
BUV395				Ki-67
BUV737				PD-1
